# Distribution patterns and industry planning of commonly used traditional Chinese medicinal plants in China

**DOI:** 10.1016/j.pld.2021.11.003

**Published:** 2021-11-19

**Authors:** Zhang-Jian Shan, Jian-Fei Ye, Da-Cheng Hao, Pei-Gen Xiao, Zhi-Duan Chen, An-Ming Lu

**Affiliations:** a State Key Laboratory of Systematic and Evolutionary Botany, Institute of Botany, Chinese Academy of Sciences, Beijing 100093, China; b University of Chinese Academy of Sciences, Beijing 100049, China; c China National Botanical Garden, Beijing 100093, China; d Biotechnology Institute, School of Environment and Chemical Engineering, Dalian Jiaotong University, Dalian 116028, China; e Institute of Medicinal Plant Development, Chinese Academy of Medical Sciences, Beijing 100193, China

**Keywords:** Medicinal plant, Distribution pattern, Traditional Chinese medicine industry, Thiessen polygon

## Abstract

Medicinal plants are the primary material basis for disease prevention and treatment in traditional Chinese medicine (TCM). The conservation and sustainable utilization of these medicinal plants is critical for the development of the TCM industry. However, wild medicinal plant resources have sharply declined in recent decades. To ameliorate the shortage of medicinal plant resources, it is essential to explore the development potential of the TCM industry in different geographical regions. For this purpose, we examined the spatial distribution of commonly used medicinal plants in China, the number of Chinese medicinal material markets, and the number of TCM decoction piece enterprises. Specifically, multispecies superimposition analysis and Thiessen polygons were used to reveal the optimal range for planting bulk medicinal plants and the ideal regions for building Chinese medicinal material markets, respectively. Furthermore, we quantitatively analyzed mismatches between the spatial distribution of commonly used medicinal plant richness, Chinese medicinal material markets, and TCM decoction piece enterprises. We found that the areas suitable for growing commonly used medicinal plants in China were mainly distributed in Hengduan Mountain, Nanling Mountain, Wuling Mountain, and Daba Mountain areas. The Thiessen polygon network based on Chinese medicinal material market localities showed there are currently fewer markets in southwestern, northwestern, and northeastern China than in central and southern China. TCM decoction piece enterprises are concentrated in a few provinces, such as Hebei and Jiangxi. We found that the distribution of commonly used medicinal plants, Chinese medicinal material markets and TCM decoction piece enterprises are mismatched in Henan, Shaanxi, Hunan, Hubei, Zhejiang, Fujian, Chongqing, and Xizang. We recommend strengthening development of the TCM industry in Henan, Hunan, Zhejiang, Shaanxi, Hubei, Chongqing, Fujian, and Xizang; building more Chinese medicinal material markets in southwestern, northwestern, and northeastern China; and establishing medicinal plant nurseries in resource-rich provinces to better protect and domesticate local medicinal plants.

## Highlights

• Areas suitable for growing commonly used medicinal plants in China are the Hengduan, Nanling, Wuling, and Daba Mountains.
• Southwestern, northwestern, and northeastern China had fewer markets than central China.
• The TCM decoction piece enterprises are most concentrated in a few provinces, such as Hebei and Jiangxi.

## 1 Introduction

Traditional Chinese medicine (TCM) is an essential part of China's medical and healthcare system because of its low cost and unique preventive/curative effects (Zhang and Chen, 2016). In TCM, medicinal plants are the primary material basis for disease prevention and treatment. The conservation and sustainable utilization of these medicinal plants are critical for the development of the TCM industry, as approximately 70% of medicinal plants in China are harvested from wild resources (Huang et al., 2012). Over 600 medicinal species are listed as Critically Endangered, Endangered, or Vulnerable in China (Chi et al., 2017). Moreover, *Panax notoginseng* (Burkill) F.H. Chen ex C. Chow & W.G. Huang has been assessed as Extinct in the Wild (Qin et al., 2017). The serious shortage in medicinal plant resources has been caused by several factors, including unsustainable harvesting practices, global warming, habitat loss and fragmentation, and land transfer (Yang et al., 2000; Marshall and Hawthorne, 2012; Chi et al., 2017).

Medicinal plant scarcity may potentially be alleviated through various approaches, including tissue culture, artificial cultivation techniques, and the use of substitutes (Larsen and Olsen, 2007; Chen et al., 2016). Among these approaches, artificial cultivation of medicinal plants is the most feasible. The high economic returns of artificially cultivating medicinal plants have attracted great interest from traditional crop farmers (Negi et al., 2018). However, most farmers do not understand the requisite cultivation practices; consequently, medicinal plants have been introduced into areas that are unsuitable for cultivation and active ingredient content of many medicinal plants cultivated in this manner has been substandard. Several researchers have proposed that the stability and controllability of Chinese medicinal material quality can be maintained through medicinal plant tending, in which a population of a target species is naturally and manually increased in its native or similar habitat. Wild medicinal plant tending is thought to provide high-quality genuine medicinal materials and effectively save costs (Chen et al., 2004; Yu et al., 2019). However, wild medicinal plant tending requires a thorough understanding of the distribution of medicinal plants in the wild.

Species occurrence data have been widely used to estimate species diversity and distribution (Maldonado et al., 2015), monitor invasive species (Dong et al., 2012), identify priority conservation species (Rivers et al., 2010), and propose suitable ecological areas for medicinal plants (Zhang et al., 2017). These data can also be used to guide wild medicinal plant tending. For example, a previous study inferred the most ecologically suitable production areas for *Panax ginseng* C.A. Meyer worldwide using ecological similarity analysis (Shen et al., 2016). Plant native occurrence data have become an effective basis for researchers to plan medicinal plant production (Luo et al., 2020). However, to date research on wild medicinal plant tending has only focused on specific medicinal species (Sun et al., 2009; Jiang et al., 2016; Yan et al., 2016). Regional or national plans that incorporate an overlay analysis of all medicinal plants have yet to be developed (Zhu et al., 2014).

Cultivated medicinal plants are usually sold by farmers to the Chinese medicinal material (CMM) markets and then circulate from the markets to the TCM decoction piece enterprises (TDPEs). Currently, there are 17 legal, specialized CMM markets in China. By gathering all types of medicinal plants and preliminarily processed products in the region, the CMM markets spread diverse resources for the TCM industry. These markets manage the planting activities of medicinal plants in the region through price adjustment. The products of TDPEs are TCM decoction pieces, which are directly supplied to clinical prescription and TCM processing enterprises. The CMM markets and TDPEs are the key links in the TCM industrial chain. Thiessen polygons can quantify the scope of raw materials sold or purchased by CMM markets or TDPEs (Schulman et al., 2007; Schlicht et al., 2014). Generally, farmers are more likely to sell their medicinal plants to the nearest CMM market, disregarding, of course, price, traffic conditions, or other factors; moreover, TDPEs are more likely to buy raw materials from the nearest CMM market.

The aim of this study is to guide regional plans for planting medicinal plants. For this purpose, we first explored the overall distribution of commonly used medicinal plants growing in the wild in China. We also explored the layout of CMM markets and TDPEs with Thiessen polygons to identify areas that lack sufficient overlap between medicinal plant resources, markets, and enterprises. Our findings allow us to make recommendations for the future development of the TCM industry.

## 2 Material and methods

### 2.1 Checklist of commonly used TCM plants

We identified *ca*. 2700 medicinal plants listed by Zhao (2017) as commonly used medicinal plants. Among them, exotic species were excluded and species names were standardized following *Flora of China* (Wu et al., 1994–2013). For consistency and comparability, only species-level taxa were retained for analysis, and infraspecies taxa were merged with the relevant species. In total, 2471 species of commonly used medicinal plants belonging to 1008 genera and 200 families were used for analysis.

### 2.2 Grid mapping of commonly used medicinal plants

In total, 753,182 occurrence records of 2305 species were collected, which accounted for 93.28% of all commonly used medicinal plants in China (Table S1). The occurrence data were mainly obtained from two sources: specimen records in the Chinese Virtual Herbarium (http://www.cvh.ac.cn/) and Lu et al. (2018).

TCM experts have suggested that some medicinal plant varieties from specific regions exhibit higher clinical efficacy, and call these varieties genuine medicinal materials (geoherb; Xiao et al., 2009). Therefore, we collected medicinal plants with “genuine area” records, removing the remaining records. In total, “genuine areas” for 371 medicinal plants were collected. The genuine medicinal material list and genuine-producing area data were obtained from the Genuine Medicinal Materials Atlas (Wang and Xu, 2003).

To convert all geographical occurrence information to grid cells, we first mapped the point occurrence to a map of China, then extracted the county-level administrative units of each point. These records were integrated with the county occurrence records obtained from Lu et al. (2018) and duplicate records were removed. Then we created a Fishnet 100 × 100 km^2^ with the map of China as the boundary. Grid cells with less than 50% area within national borders and coastal regions were removed, which resulted in a total of 943 grid cells. After matching counties and grids, we generated a species-level presence–absence matrix of commonly used medicinal plants for these 943 grid cells. These analyses above were conducted in ArcGIS 10.0.

### 2.3 Distribution of TCM decoction piece enterprises and Chinese medicinal material markets

Data on distribution of TDPEs were collected from the National Medical Products Administration (NMPA) (http://app1.nmpa.gov.cn/). We selected drug manufacturing enterprises, searching TCM decoction pieces as a qualifier. All the retrieved data were stored in EXCEL for analysis (accessed 01 Dec. 2020). We used all the available provincial administrative units in mainland China (31) for TDPE in NMPA in the following analysis. The CMM market locations were obtained from 17 large CMM professional markets approved by the central government of the People's Republic of China.

### 2.4 Thiessen polygons

The Thiessen polygon is an algorithm based on analyzing spatial proximity. Each Thiessen polygon contains the data of only one discrete point; any point within a Thiessen polygon is closest to the sample point of the polygon, and points on the edge of a Thiessen polygon are equidistant from discrete points on both sides (Aurenhammer et al., 2013). As the NMPA lacked accurate location information for TDPEs, we used the positions of professional CMM markets only to draw the Thiessen polygons with ArcGIS 10.0. First, we imported the points of 17 CMM markets into ArcGIS 10.0 and selected Analysis Tools in ArcToolbox. Subsequently, we selected Proximity in the Analysis Tools and Create Thiessen Polygons. Finally, we set the boundary of the Thiessen polygon as the border of China. To compare the coverage area of CMM markets in different regions, we divided Thiessen polygons into two groups: group 1 was distributed in central and southern China, and group 2 was distributed in southwestern, northwestern, and northeastern China (Table 2). Then the area of these two groups was used for the Student's t-test.

## 3 Results

### 3.1 Geographic distribution pattern of commonly used medicinal plants

**Fig. 1:**
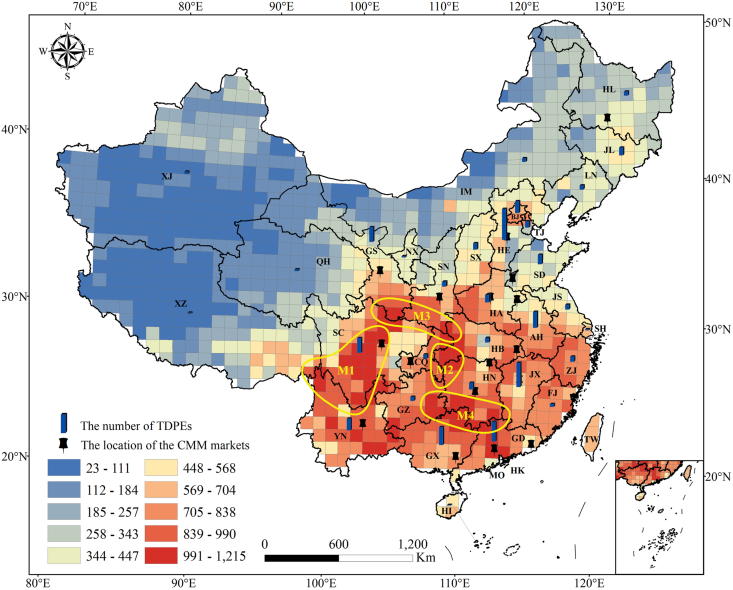
Spatial patterns of all commonly used medicinal plants, traditional Chinese medicine decoction piece enterprises (TDPEs) and Chinese medicinal material (CMM) markets. Blue columns represent TDPEs and their number (height) in each province. Black pins represent the locations of CMM markets. M1: the Hengduan Mountain area; M2: the Wuling Mountain area; M3: the Daba Mountain area; M4: the Nanling Mountain area; AH: Anhui; BJ: Beijing; CQ: Chongqing; FJ: Fujian; GD: Guangdong; GS: Gansu; GX: Guangxi; GZ: Guizhou; HA: Henan; HB: Hubei; HE: Hebei; HI: Hainan; HK: Hong Kong; HL: Heilongjiang; HN: Hunan; IM: Inner Mongolia; JL: Jilin; JS: Jiangsu; JX: Jiangxi; LN: Liaoning; MO: Macao; NX: Ningxia; QH: Qinghai; SC: Sichuan; SD: Shandong; SH: Shanghai; SN: Shaanxi; SX: Shanxi; TJ: Tianjing; TW: Taiwan; XJ: Xinjiang; XZ: Xizang; YN: Yunnan; ZJ: Zhejiang.

Commonly used medicinal plants were distributed in all grid cells, with an average of 411.7 per grid cell. Medicinal plant diversity was higher in southern than in northern China. In general, these medicinal plants were concentrated in the following four distribution centers: 1) the Hengduan Mountain area, including western Sichuan, northwestern Yunnan, and eastern Xizang; 2) the Wuling Mountain area, crossing the borders of Hubei, Hunan, Guizhou, and Chongqing; 3) the Daba Mountain area, spanning the borders of Sichuan, Gansu, Shaanxi, and Hubei; 4) the Nanling Mountain area, i.e. the borders of Jiangxi, Hunan, Guizhou, Guangxi, and Guangdong (Fig. 1).

### 3.2 Distribution of TCM decoction piece enterprises

In our study, a total of 2465 TDPEs were examined from 31 provincial-level administrative regions. On average, there were 79.5 TDPEs in each province. The distribution of these TDPEs was extremely uneven. The number of TDPEs exceeded the average in only 10 provinces, e.g., the top three included Hebei (296), Jiangxi (233), and Guangdong (190); these provinces accounted for 66.77% of the total number of TDPEs (Table 1). In contrast, the number of TDPEs was much lower than average in Hainan (7), Xizang (10), Shanghai (11), Ningxia (13), and Qinghai (19) (Table 1).

**Table 1:** Numbers of TCM decoction piece enterprises (TDPEs), commonly used medicinal plants, and Chinese medicinal material (CMM) markets in each province.

Province of China	Number of TDPEs	Number of commonly used medicinal plants	Number of CMM markets
Hebei	296	854	1
Jiangxi	233	1112	1
Guangdong	190	1377	2
Guangxi	173	1502	1
Anhui	154	900	1
Sichuan	143	1818	1
Gansu	141	1179	1
Yunnan	113	1741	1
Beijing	113	718	0
Shandong	90	548	1
Jilin	74	537	0
Henan^a^	72	1073	1
Hunan^a^	64	1276	2
Tianjin	61	364	0
Shanxi	60	837	0
Zhejiang^a^	49	1047	0
Shaanxi^a^	48	1189	1
Jiangsu	46	769	0
Hubei^a^	43	1251	1
Chongqing^a^	41	1234	1
Liaoning	39	625	0
Heilongjiang	39	476	1
Guizhou	38	1398	0
Inner Mongolia	34	831	0
Fujian^a^	28	1200	0
Xinjiang	23	526	0
Qinghai	19	703	0
Ningxia	13	460	0
Shanghai	11	605	0
Xizang^a^	10	1026	0
Hainan	7	693	0

a These provinces in particular need to strengthen TCM industry development.

### 3.3 Mismatches in the distributions of medicinal plant resources, Chinese medicinal material markets, and TCM decoction piece enterprises

There were 17 CMM markets in 15 provinces. These markets were mainly concentrated in central and southern China. Among the provinces, Guangdong and Hunan each had two CMM markets, whereas Anhui, Sichuan, Henan, Jiangxi, Chongqing, Heilongjiang, Gansu, Shaanxi, Hubei, Guangxi, and Yunnan each had one CMM market (Fig. 1).

**Fig. 2:**
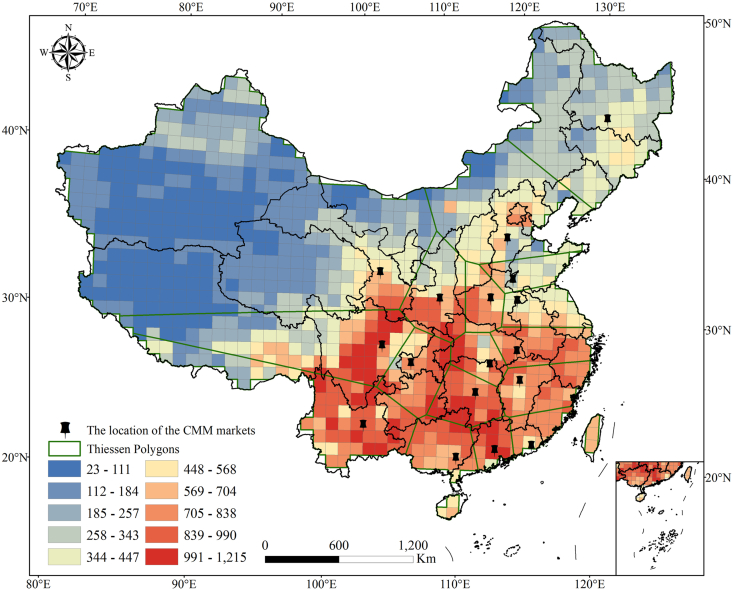
The Thiessen polygon network based on Chinese medicinal material (CMM) markets. The green polygons represent the Thiessen polygons, and the black pins represent the location of CMM markets.

The Thiessen polygon network based on CMM market localities visualized the extent of land covered by a single CMM market (Fig. 2). The polygons showed that the area overlaid by a CMM market was significantly larger in southwestern, northwestern, and northeastern China than that in central and southern China (*p* < 0.01) (Fig. 2). Lanzhou Huanghe CMM market of Gansu Province covered the largest area (i.e., 3,542,534.10 km^2^). There are five polygons with an area of more than 500,000 km^2^ (Table 2). Notably, the Qingping CMM market of Guangzhou covers the smallest area (109,039.2 km^2^), which is 32.5 times smaller than the largest one (Table 2).

**Table 2:** Area of Thiessen polygons covered by Chinese medicinal material (CMM) markets.

Name	Province	Area (km^2^)	Group
Guangzhou Qingping CMM market	Guangdong	109,039.2	1
Guangdong Puning CMM market	Guangdong	133,382.1	1
Hunan Yueyang Huabanqiao CMM market	Hunan	137,143.3	1
Shandong Shunwangcheng CMM market	Shandong	143,860.2	1
Henan Yuzhou CMM market	Henan	145,104.9	1
Anhui Bozhou CMM market	Anhui	184,281.1	1
Hunan Shaodong CMM market	Hunan	201,807.3	1
Hubei Qizhou CMM market	Hubei	219,046.3	1
Guangxi Yulin CMM market	Guangxi	230,171.4	1
Jiangxi Zhangshu CMM market	Jiangxi	239,295.4	1
Chongqing Jiefanglu CMM market	Chongqing	260,412.6	1
Xian Wanshoulu CMM market	Shaanxi	311,988.3	2
Kunming Juhuayuan CMM market	Yunnan	640,039.9	2
Hebei Anguo CMM market	Hebei	746,679.4	2
Chengdu Hehuachi CMM market	Sichuan	842,567.6	2
Harbin Sankeshu CMM market	Heilongjiang	1,132,438.7	2
Lanzhou Huanghe CMM market	Gansu	3,542,534.1	2

## 4 Discussion

### 4.1 Uneven distribution of commonly used medicinal plants

In this study, we explored the distribution of commonly used medicinal plants in China and found that the medicinal plant richness is concentrated in four mountain areas (Fig. 1). The Hengduan Mountain area has a complex terrain and climate; for alpine plants in China, this mountain area serves as a refuge and a place of origin (Ding et al., 2020). Representative medicinal plants in this area include *Fritillaria cirrhosa* D. Don, *Angelica sinensis* (Oliv.) Diels, and *Rheum palmatum* L. (China National Chinese Medicinal Material Corporation, 1995). The Wuling Mountain area has typical karst habitat and is one of three distribution centers of endemic plant genera in China (Ying, 2001). This area has many genuine medicinal plants, such as *Fallopia multiflora* (Thunb.) Harald., *Pseudostellaria heterophylla* (Miq.) Pax, and *Coptis chinensis* Franch. The Daba Mountain area has a moderate climate with abundant sunlight, rainfall, and heat, and has an added advantage of diverse ecological conditions for growing medicinal plants (Zhang et al., 2008). Representative medicinal plants in this area are *Eucommia ulmoides* Oliver, *Cornus officinalis* Sieb. et Zucc., and *Ligusticum sinense* Oliver (China National Chinese Medicinal Material Corporation, 1995). Finally, most parts of the Nanling Mountain area belong to a series of hilly basins with red rocks, and the medicinal plants there grow in acidic soil. A representative medicinal plant in this area is *Houpoea officinalis* (Rehder & E.H. Wilson) N.H. Xia & C.Y. Wu. One reason for the high abundance of medicinal plants in these areas may climate and habitat heterogeneity and patterns of the overall plant species diversity (Tang et al., 2006; Hu et al., 2020). Interestingly, the distribution of the most commonly used medicinal plants, endemic medicinal plants (Li et al., 2017), and threatened medicinal plants (Chi et al., 2017) overlap. Wild tending of threatened and endemic medicinal plants in these hotspots will promote the protection of these species.

In addition, these distribution centers are characterized by low economic development. For example, the Wuling Mountain area is one of the original 14 contiguous impoverished areas in China (Huang et al., 2017). The complex topographical conditions in these areas have historically impeded industrial development. Wild medicinal plant tending in mountain areas is a promising approach to develop local economies. The development of medicinal plant cultivation will provide income for local people engaged in various aspects of harvesting, processing, and marketing (Laird et al., 2010). In contrast to these mountain areas, crops such as rice are planted in the plain areas. The intercropping of food crops and medicinal plants can be carried out in suitable areas, which will both increase economic benefits and promote the full use of land resources (Jiang et al., 2017).

### 4.2 Optimizing the layout of TCM decoction piece enterprises and Chinese medicinal material markets

TDPEs were mainly distributed in Hebei, Jiangxi, Guangdong, Guangxi, and Anhui (Fig. 1). TCM decoction piece processing is a traditional resource-dependent industry, and the source of raw material is the main bottleneck in enterprise development. Regional medicinal plant resources are gathered in CMM markets, which provide a stable source of raw materials for TDPEs to maintain continuous production. However, the medicinal species diversity of a region is not always commensurate with the number of CMM markets or TDPEs (Table 1). For example, in Hunan, Hubei, Chongqing, and Shaanxi over 1000 commonly used medicinal plants occur. Although each of these provincial units has one CMM market, the number of TDPEs is lower than the provincial average (79.5) (Table 1). In addition, Guizhou, Fujian, and Xizang are rich in commonly used medicinal plants, but there are no authorized CMM markets in these provinces, and development of the TCM industry is relatively weak.

The Thiessen polygons of CMM markets in southwestern, northwestern, and northeastern China were larger than those in the central and southern provinces (Table 2). The larger the area covered by the CMM markets, the higher the average cost for farmers and enterprises to sell or purchase medicinal herbs. One potential explanation for why CMM markets are large in some regions is a lack of infrastructure (e.g., transportation), which hinders CMM market development. In addition, there are many contiguous regions without a CMM market, most notably in northwestern China (Fig. 2). This region produces most traditional Tibetan medicine (Hao et al., 2018), and many valuable medicinal plants are endemic to this region, such as *Rhodiola crenulata* (Hook. f. et Thoms.) H. Ohba and *Neopicrorhiza scrophulariiflora* (Pennell) D.Y. Hong. The lack of a CMM market has greatly impeded the sales of medicinal plant products by farmers and the acquisition of pharmaceutical raw materials by TDPEs in this region, which partially explains the lagging development of the local TCM industry. Therefore, we recommend government agencies that regulate the development of the TCM industry focus on areas where commonly used medicinal plants are abundant but TDPEs are insufficient (e.g., Hunan, Hubei, Chongqing, and Shaanxi) (Table 1).

### 4.3 Implications for TCM industry development

Our study determined that the distribution patterns of commonly used medicinal plants, CMM markets, and TDPEs in China are mismatched in Henan, Shaanxi, Hunan, Hubei, Zhejiang, Fujian, Chongqing, and Xizang (Table 1). Each of these provinces is capable of growing more than 1000 commonly used medicinal plants, but the number of TDPEs is below average. In the future, the government can guide these provinces to plant medicinal plants and support the development of TDPEs, which will improve the livelihood of local residents. It is gratifying that some of these provinces have begun to take action. For example, Zhejiang Province has focused on eight representative genuine medicinal materials and reformulated their strategy for TCM industry development.

There are 17 CMM markets in China, and the coverage of these markets varies greatly (Fig. 1). In the past, CMM markets were established due to historical factors, which neglected local resources (Zhou, 2016). Consequently, CMM markets have had difficulty adapting to the rapid development of the current TCM industry. For example, the major manufacturers (TDPE in Xizang) of Qishiwei Zhenzhu pills, Tibet Ganlu TMM Co., Ltd. and Jinhe TMM Co., Ltd., have obtained three approval numbers, and the annual sales are expected to reach approximately 400 million yuan in 2020 (Fu et al., 2020). However, to date, there has been no official CMM market in Xizang. To a certain extent, this has increased the cost of purchasing raw materials for pharmaceutical companies. New CMM markets should be established in provinces with abundant medicinal resources.

The adoption of Good Agricultural Practice (GAP) protocols have increased the number of TDPEs located near the source of their raw materials, i.e., where medicinal materials originate. However, it is difficult for TCM enterprises to establish GAP bases without records of areas that produce genuine medicinal plants. This study identifies regions suitable for growing specific Chinese herbal medicine (CHM) plants, providing an important reference for farmers, enterprises and government agencies. This guidance can both prevent the development of homogenization in different regions and facilitate macro-control of CHM prices to promote the healthy development of the TCM industry. In addition, the protection of wild medicinal plants and the cultivation of high-yield new varieties of medicinal plants are also extremely important for the sustainable development of the TCM industry (Wei et al., 2011; Chi et al., 2017). Because only approximately 300 medicinal plants are under large-scale cultivation (Chen and Xiao, 2006), some provinces with abundant medicinal plants can be selected to establish medicinal plants nurseries to supply quality planting materials and promote the cultivation of threatened high-value species. For example, a nursery to preserve Tibetan medicinal plants could be established in Nyingchi, Xizang.

## 5 Conclusions

The cultivation, sale, and processing of medicinal plants are key links in the TCM industry chain. This is the first study to examine the distribution of medicinal plants, Chinese medicinal material markets, and TCM decoction piece enterprises. We determined that 1) four mountain areas with the highest medicinal plant diversity in China are important for wild medicinal plant tending; 2) the government should provide additional financial and technical support for the TCM industry in Henan, Hunan, Zhejiang, Shaanxi, Hubei, Chongqing, Fujian, and Xizang to match the medicinal plant richness of these regions; 3) the government should build more CMM markets in southwestern, northwestern, and northeastern China to strengthen the commodity circulation of TCM and ensure CMM quality, which will help develop local TCM industry. In contrast to previous industry planning for a single species, our study overlaid the areas suitable for diverse medicinal plants. Multispecies superimposition analysis has strong practicability and can provide a realistic reference for local governments to formulate development plans for local TCM industry.

## Availability of data and material

All data generated and analyzed during this study are included in this published article and its supplementary information files.

## Author contributions

J.F.Y., Z.D.C. conceived the study; Z.J.S. conducted the analyses; Z.J.S., J.F.Y., D.C.H., P.G.X., Z.D.C. and A.M.L. wrote the manuscript; all authors read and approved the final manuscript.

## Declaration of competing interest

The authors declare that they have no conflict of interest.
